# A preliminary study on the reference intervals of vitamin K in some areas of Beijing with normal physical examination population

**DOI:** 10.1017/jns.2025.1

**Published:** 2025-02-05

**Authors:** Lin Chen, Meiqi Chen, Shi Cheng, Jiaxin Fei, Dan Xu, Xueyun Hou, Nannan Li, Yuliang Yuan, Guijian Liu, Cheng An

**Affiliations:** 1 Clinical Laboratory, Guang’anmen Hospital, China Academy of Chinese Medical Sciences, Beijing, China; 2 Clinical Laboratory, Guang’anmen Hospital, South District, China Academy of Chinese Medical Sciences, Beijing, China

**Keywords:** Apparent normal population, Nutritional status, Reference interval, Serum level, Vitamin K, VK1, Vitamin K1, MK4, Menaquinone-4, MK7, Menaquinone-7, ng/ml, nanograms per millilitre, BMI, body mass index, Hb, haemoglobin, FG, fasting glucose, Cr, creatinine, UA, uric acid, ALT, alanine aminotransferase, AST, aspartate aminotransferase, TC, cholesterol, TG, triglycerides, HDL-C, high-density lipoprotein cholesterol, LDL-C, low-density lipoprotein cholesterol

## Abstract

The vitamin K (VK) levels vary greatly among different populations and in different regions. Currently, there is a lack of reference intervals for VK levels in healthy individuals, The aim of this study is to establish and validate the reference intervals of serum vitamin K1 (VK1) and vitamin K2 (VK2, specifically including menaquinone-4 (MK4) and menaquinone-7 (MK7)) levels in some healthy populations in Beijing. Serum VK1, MK4, and MK7 were firstly measured by high-performance liquid chromatography and mass spectrometry in 434 subjects. The reference intervals for three indicators were established by calculating the data of 2.5 and 97.5 percentiles. Finally, preliminary clinical validation was conducted on 60 apparent healthy individuals undergoing physical examination. In the young, middle-aged, and elderly groups, the reference intervals of VK1 were 0.180 ng/mL ∼ 1.494 ng/mL, 0.247 ng/mL ∼ 1.446 ng/mL, and 0.167 ng/mL ∼ 1.445 ng/mL, respectively. The reference intervals of MK4 were 0.009 ng/mL ∼ 0.115 ng/mL, 0.002 ng/mL ∼ 0.103 ng/mL, and 0.003 ng/mL ∼ 0.106 ng/mL, respectively. The reference intervals of MK7 were 0.169 ng/mL ∼ 0.881 ng/mL, 0.238 ng/mL ∼ 0.936 ng/mL, and 0.213 ng/mL ∼ 1.012 ng/mL, respectively. The reference intervals had been validated by the samples of healthy individuals for physical examination. In conclusion, the reference intervals of VK established in this study with different age groups have certain clinical applicability, providing data support for further multicentre studies.

Vitamin K (VK) is an essential nutritional component of the human body, involved in various physiological and pathological functions. It mainly differentiates a series of compounds into Vitamin K1(VK1) and Vitamin K2 (VK2) based on their structures. VK1 is mainly stored in the liver, and is crucial for the activation of coagulation factors and the maintenance of calcium homeostasis.^(1)^ VK2, also known as menaquinone-4 (MK-4) and menaquinone-7 (MK-7), is a type of vitamin K that contains a varying number of isoprene units (n). It includes variants from MK4 to menaquinone-15(MK15). These variants are widely distributed throughout the circulation, liver, and extrahepatic tissues. They have been linked to various health issues such as osteoporosis, vascular calcification, osteoarthritis, cancer, and cognitive impairment. A deficiency or inadequacy of VK can lead to compromised physiological health, which manifests clinically as bleeding disorders. Conversely, excessive intake may result in accumulation and potential adverse health effects.^(2–4)^ Therefore, accurately assessing VK nutritional status in different populations is essential.

Despite recent efforts by the Chinese National Health Commission to establish reference intervals for various clinical test indicators, specific guidelines for VK are still lacking. There is a lack of research on VK serum levels both domestically and internationally. Factors that affect VK levels include ethnicity, body mass index (BMI), dietary preferences, and lifestyle habits. Notably, VK levels can vary greatly within populations; for instance, postmenopausal women in Tokyo, Japan, have a reported VK2 level of 5.26 ± 6.13 ng/mL, while the same demographic in western Japan shows a level of just 1.22 ± 1.85 ng/mL.^(5)^ This highlights the need for evaluating VK status among specific populations in Beijing and developing corresponding biological reference intervals.

Furthermore, VK is crucial for the synthesis and activation of coagulation factors and maintaining calcium balance, and its link with conditions like osteoporosis, vascular calcification, cancer, and cognitive impairment is well documented.^(2–4)^ Unfortunately, many people, especially in China, have insufficient VK intake, resulting in regional differences in reference intervals. Clinically, VK deficiency poses more common and serious risks than excess, as shown by conditions such as spontaneous skin purpura, nosebleeds, and other bleeding disorders. The VK levels in different groups are affected by various factors, including regional and dietary habits, which makes the use of inappropriate biological reference intervals particularly worrying. Such misuses could result in misdiagnosis, delayed treatment, unnecessary medical costs, and increased risks for patients.

Current studies on VK levels and reference intervals in China mainly focus on specific groups such as pregnant women, the elderly, children, and individuals with coronary heart disease.^(6–9)^ However, there is a significant lack of research on VN levels among healthy individuals. Most available references are derived from international data, as VK clinical bioanalysis—particularly at low concentrations—remains challenging due to its lipophilic nature and scarcity.^(10)^ Clinical evaluation of VK levels typically depends on two methodologies: direct serum or plasma VK level measurements, which often employ liquid chromatography coupled with mass spectrometry, but may be influenced by factors like lipemia; and indirect methods assessing VK-dependent proteins (VKDP), such as undercarboxylated osteocalcin (ucOC), underphosphorylated carboxylated matrix Gla protein (dp-ucMGP), and proprotein induced by vitamin K deficiency II (PIVKA-II). However, these methodologies lack standardization and are variably affected by different physiological and pathological states.^(10,11)^ Currently, a standardised reference interval for these indicators is absent, and reported reference intervals for diverse populations or physiological states are limited.^(5,10)^ Therefore, setting up reference intervals for VK assessment indicators is essential for optimizing clinical diagnostic and therapeutic services.

In this study, we aim to: 1. Comprehensively investigate and elucidate the disparities in serum VK concentration distribution across diverse genders and age demographics within select regions of Beijing.; 2. Establish robust and statistically valid reference intervals for serum VK levels, ensuring its applicability and reliability across the studied population. 3. Perform a preliminary clinical validation of the established reference intervals, assessing its utility and accuracy in a real-world clinical setting.

## Study design

In this cross-sectional study, we employed random sampling to select healthy individuals undergoing routine health examinations. Participant recruitment was based on a comprehensive assessment that included questionnaire surveys, physical examinations, and laboratory screenings. We adhered strictly to the inclusion and exclusion criteria to determine the final cohort of study subjects. The study population was concurrently evaluated for VK1, MK4, and MK7 levels and stratified into different age groups, with each group further divided into gender subgroups. After the exclusion of outliers within each group, we analysed the differences between and among these groups and subgroups. Groups lacking statistical differences did not have reference intervals calculated separately, whereas those with significant statistical differences had their reference intervals calculated individually. Following the establishment of reference intervals for each group, we selected a new set of healthy subjects for preliminary clinical validation. This approach allowed us to assess the applicability and reliability of the reference intervals in a real-world context, ensuring that our findings are robust and reflect the diverse characteristics of the study participants.

## Methods

Subjects: All subjects were selected from apparently healthy individuals who underwent physical examinations at Guang’anmen Hospital, China Academy of Chinese Medical Sciences, From 2019 to 2023. All the samples were selected using the simple random sampling method. According to the recommendation of the Clinical and Laboratory Standards Institute (CLSI), a professional organization that develops guidelines for clinical laboratories, in its document C28-A3 ‘Defining, Establishing, and Verifying Reference In Clinical Laboratory; Approved Guideline-Third Edition’, a minimum of 153 cases is required to estimate reference limits with a 95% confidence interval, while 198 cases are necessary for a 99% confidence interval. Given the wide age interval of adults and the potential need for age stratification, a sample size of at least 300 adults was considered necessary. Initially, 2113 apparently healthy subjects, between 18 and 84 years, were selected in this study. Subsequently, 434 healthy individuals with all the following test indicators within normal intervals were ultimately included in this study. The inclusion criteria in conjunction with the Chinese clinical reference interval were as follows: body mass index (BMI, 18.5–24 kg/m^2^), haemoglobin (HB, 115–150 g/L for females and 120–160 g/L for males), fasting glucose (FG, 3.9–6.1 mmol/L), urea (UREA, 2.5–7.5 mmol/L), creatinine (Cr, 44–133 μmol/L for females and 53–106 μmol/L for males), uric acid (UA, 140–420 μmol/L), alanine aminotransferase (ALT, 5–40 U/L), aspartate aminotransferase (AST, 8–40 U/L), cholesterol (TC, <5.2 mmol/L), triglycerides (TG, <1.7 mmol/L), high-density lipoprotein cholesterol (HDL-C, ≥1.04 mmol/L), and low-density lipoprotein cholesterol (LDL-C, <3.4 mmol/L). Subjects with one of the following conditions or diseases were excluded: use of antibiotics for more than 10 days, digestive system diseases, long-term diarrhoea, liver disease, jaundice, liver dysfunction, pancreatic dysfunction, diabetes, renal dysfunction, family history of bleeding and coagulation related diseases, anaemia, taking oestrogen drugs, taking lipid-lowering or weight loss drugs, recent vitamin supplementation, nervous system diseases, immune system diseases, tumours, abnormal blood pressure, coronary heart disease, atherosclerosis, and dyslipidaemia. This study was approved by the Institutional Review Board of Guang’anmen Hospital, China Academy of Chinese Medical Sciences (approval number: 2020-017-KY). All participants provided written informed consent, and their confidentiality and anonymity were ensured.

Questionnaire survey: including data on basic information (such as gender, age, ethnicity, and occupation), recent personal medication history (such as whether they use vitamins, phenytoin sodium, antibiotics, weight-loss drugs, hormone drugs, bile acid sequestrants, health products, and drugs containing vitamin K1 and vitamin K2), family medical history (including whether there are familial bleeding and coagulation-related diseases), dietary habits and whether they have smoking and drinking habits.

Physical examination: BMI was calculated from height and weight that were measured by unified method and equipment. Fasting venous blood samples were centrifuged at 1500 X g for 15 min, 20–30 min after being collected. All blood samples were frozen at -80°C for subsequent detection and analysis. Hb were determined using an automated haematology analyser (Sysmex XT-2000i, Kobe, Japan). Total cholesterol, TG, LDL-C, HDL-C, FG, Urea, Cr, UA, ALT, AST were measured enzymatically using an automated biochemistry analyser (Beckman AU5800, California, American).

Baseline Characteristic Control: To objectively describe the differences in the reference interval of relevant indicators in the healthy population, the research subjects are fully controlled within the normal healthy population through the above-mentioned questionnaire survey and physical examination, combined with the application of inclusion and exclusion criteria.

VK1, MK-4, and MK-7 Detection: VK1, MK-4, and MK-7 concentrations in serum were detected using high-performance liquid chromatography-tandem mass spectrometry (LC-MS/MS) on an SPD-M30A/LC-30ADCL-MS8050CL system (Shimadzu, Japan). Isotope-labelled analogues of VK1, MK-4, and MK-7 served as internal standards, which were added to serum/plasma samples and mixed thoroughly. After liquid-liquid extraction and subsequent concentration by nitrogen drying, the samples were analysed using the mass spectrometer. Mass spectrometry detection employed an APCI ion source with positive ion mode and multiple reaction monitoring (MRM). The chromatographic separation of VK1/MK-4/MK-7 from impurities was performed on Kintex Phenyl-Hexyl and Kintex C18 columns with a gradient elution of acetonitrile and methanol containing 0.2% formic acid. Concentrations of VK1/MK-4/MK-7 in serum/plasma were ascertained by calculating the ratio of the target peak areas to the internal standard peak areas, calibrated against standard solutions of VK1/MK-4/MK-7. The laboratory implemented a standard quality control protocol, establishing low (VK1: 0.30 ng/mL; MK-4: 0.30 ng/mL; MK-7: 0.30 ng/mL), medium (VK1: 2.20 ng/mL; MK-4: 0.86 ng/mL; MK-7: 0.82 ng/mL), and high (VK1: 400.00 ng/mL; MK-4: 24.00 ng/mL; MK-7: 24.00 ng/mL) concentration levels of quality control materials. The precision and accuracy of the assay were ensured by evaluating sample repeatability, intra-laboratory precision, and the method of spiked recovery, aligning with quality management standards.

Grouping of study subjects: The participants in this study were divided into three distinct groups in accordance with the World Health Organization’s classification of human age: the young group (ages 18–44), the middle-aged group (ages 45–59), and the elderly group (ages 60 and above). Furthermore, within each age group, the subjects were further split into two subgroups based on sex: male and female.

Outlier removal: The final included research subjects underwent testing for VK1, MK4, and MK7, and the Tukey method was used to check for outliers in the detection results of the three indicators. Outliers are defined as reference values within each group that are greater than the upper interquartile interval (Q3,75% quantile)+1.5 times the interquartile interval (IQR: Q3-Q1) or less than the lower interquartile interval (Q1,25% quantile) -1.5 times the interquartile interval (IQR); For the defined outliers mentioned above, trace them back to the original database and comprehensively analyse the causes of the outliers based on screening questionnaires and their authenticity checks, physical examinations, and laboratory test results; If there is a clear reason, outliers should be excluded, otherwise outliers will not be excluded.

Calculation of reference interval: the middle 95% interval was adopted as the reference interval.^(12)^ If the data within each group exhibited normal distribution, the 95% data distribution was represented by x ± 1.96 s. If the data within each group exhibited non-normal distribution, non-parametric methods were used to calculate the 2.5th percentile and the 97.5th percentile for the actual reference sample groups. Subsequently, biological reference intervals were established based on whether there were differences among the intervals of various groups.

Verification of reference intervals: According to the requirements of ISO 15189:2022 international standard for reference interval performance verification, a reference interval requires 20 samples for verification. Therefore, 60 healthy participants were randomly selected from three age groups: young, middle-aged, and elderly, with 20 people in each group, including 10 males and 10 females, to verify the reference intervals of serum VK1, MK4, and MK7 levels in different age groups. Compare the test results of healthy participants with the established reference intervals. If no more than 10% of the detection values exceed the lower or upper limit, it is considered that the reference interval validation has passed. Otherwise, it indicates that the reference interval may not be applicable to the region, so it is necessary to increase the number of validation samples or re-establish the biological reference interval with a larger sample size.

Quality control: We implemented stringent quality control measures to ensure standardization and normalization across all stages of the process. Comprehensive Standard Operating Procedures (SOPs) were put in place, covering every aspect from sample collection and transportation to processing, storage, and quality inspection. Serum samples were promptly obtained by centrifugation within sixty minutes post-collection and subsequently stored at -80°C following separation to preserve integrity. Any specimens exhibiting haemolysis or chylomicronaemia were meticulously excluded to maintain the highest standards of data reliability.

## Statistical analyses

The data was processed and analysed using SPSS 19.0 software. The serum concentrations of all indicators are expressed in the form of P50 (P25–P75) because they do not follow a normal distribution. Kruskal–Wallis H rank sum test was used for the comparison of multiple independent samples. Two-sided P-values were calculated to assess the statistical significance of the associations. All reported *P* values are two-tailed, and *P* < 0.05 was considered statistically significant.

## Results

### Basic information

**Figure 1.**
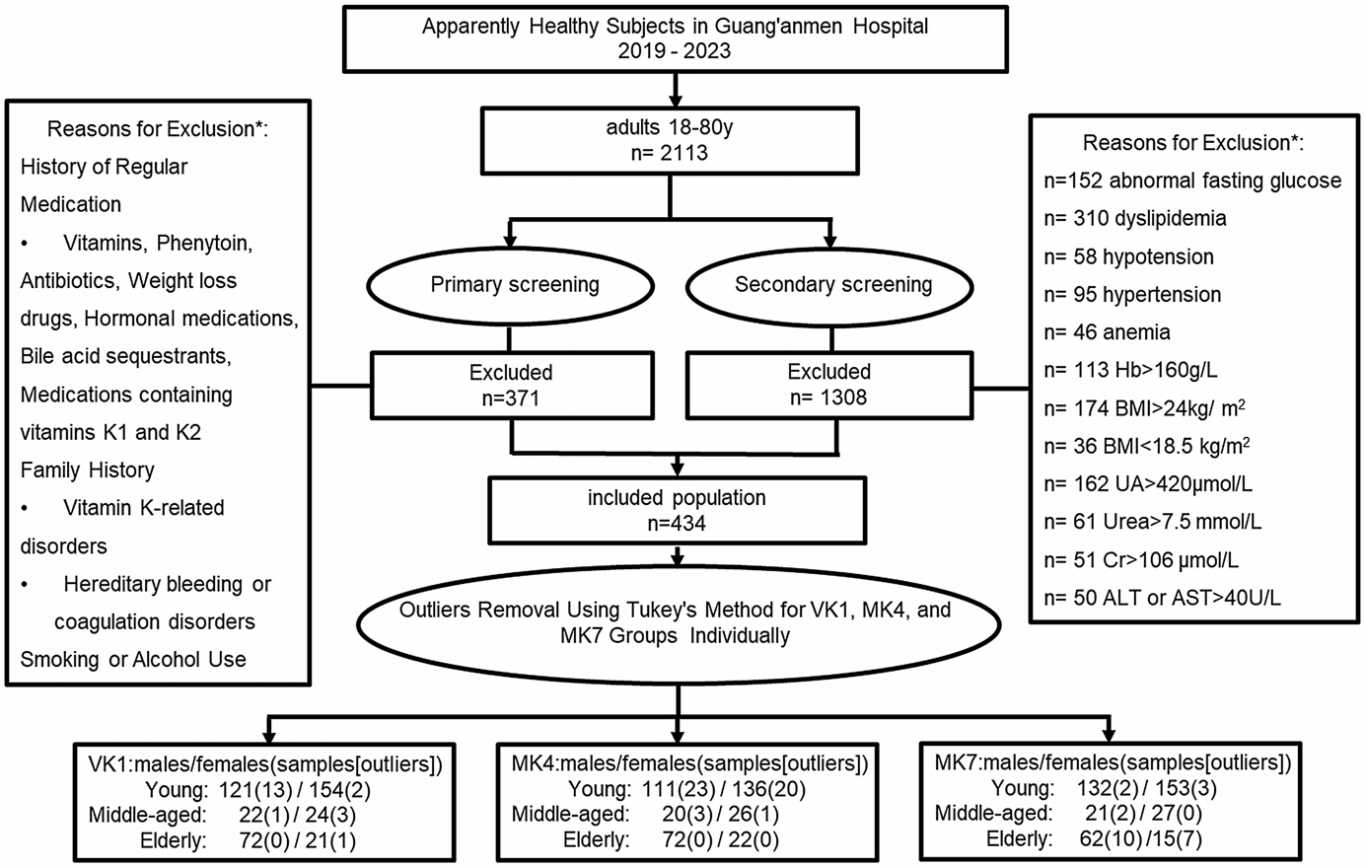
Inclusion and exclusion schematic to establish reference interval of vitamin K in some areas of Beijing with normal physical examination population; Hb, haemoglobin; BMI, body mass index; UA, uric acid; Cr, creatinine; ALT, alanine aminotransferase; AST, aspartate aminotransferase; * Healthy volunteers were obtained by sequential exclusion based on the above criteria.

After screening based on exclusion criteria, a total of 434 healthy examinees were included, with 134 males and 156 females in the young group, 23 males and 27 females in the middle-aged group, and 72 males and 22 females in the elderly group. Outlier removal was done for VK1, MK4, and MK7 to get the final research objects (Figure 1). The basic clinical data (BMI, Hb, FG, Urea, Cr, UA, ALT, AST, TC, TG, LDL-C, and HDL-C) are summarised and presented in Table 1.

**Table 1.** Basic characteristics of the study population

Characteristic	Young		Middle-aged		Elderly	
	Male	Female	Male	Female	Male	Female
BMI (kg/m2)^abc^	21.17(19.37–22.16)	21.74(20.11–22.58)	23.16(22.71–23.68)	22.66(21.81–23.22)	23.20(22.72–23.68)	22.61(21.78–23.39)
Hb (g/L) ^ab^	157.50(151.00–158.00)	132.00(125.25–140)	154.00(149.00–159.00)	133.00(123.00–136.00)	149.50(142.00–156.00)	130.00(125.00–133.50)
FG (mmol/L)^c^	5.19(4.80–5.49)^a^	4.99(4.80–5.27)^ab^	5.26(4.89–5.71)	5.08(4.76–5.77)^b^	5.03(4.60–5.84)^a ^	5.18(5.15–5.75)^ab^
Urea(mmol/L) ^abc^	4.54(3.64–5.28)	4.12(3.60–4.84)	5.07(4.76–6.20)	4.71(4.14–5.10)	4.85(3.98-5.50)	4.48(4.35–5.40)
Cr(μmol/L)^abc^	74.50(68.00–81.00)	54.00(49.00–59.00)	70.00(66.00–77.00)	59.00(51.00–64.00)	76.00(68.25–84.00)	64.00(55.00–68.25)
UA(μmol/L)^abc^	370.94(342.25–405.08)	256.50(221.00–304.00)	335.00(319.00–386.00)	290.00(247.00–326.00)	385.00(339.00–420.00)	309.00(283.00–313.00)
ALT (U/L)^c^	18.00(14.00–23.78)^ab^	11.40(8.90–15.90)^ab^	19.00(14.07–26.00)^ab^	20.10(14.00–26.20)^ab^	15.00(12.00–20.00)^b^	15.10(10.65–24.30)^b^
AST(U/L) ^abc^	20.47(18.00–24.62)	16.90(15.20–19.40)	22.00(20.00–23.00)	22.00(18.20–25.30)	21.00(18.00–25.75)	23.00(18.83–24.90)
TC (mmol/L) ^abc^	4.38(3.85–4.90)	4.34(3.99–4.66)	4.60(4.45–4.60)	4.76(4.40–5.08)	4.84(4.285–5.02)	4.53(4.37–5.11)
TG (mmol/L) ^abc^	1.00(0.72–1.29)	0.84(0.65–1.04)	1.11(1.04–1.27)	1.08(0.79–1.42)	0.96(0.79–1.24)	1.49(1.23–1.57)
HDL-C (mmol/L) ^abc^	1.14(1.04–1.27)	1.41(1.27–1.49)	1.25(1.16–1.33)	1.38(1.28–1.52)	1.17(1.04–1.32)	1.4(1.23–1.45)
LDL-C (mmol/L) ^ab^	2.53(2.06–2.93)	2.69(2.34–3.03)	2.50(0.99–2.78)	2.87(2.49–3.29)	2.55(2.15–2.82)	2.68(2.45–3.31)

BMI, body mass index; Hb, haemoglobin; FG,fasting glucose; Cr, creatinine; UA, uric acid; ALT, alanine aminotransferase; AST, aspartate aminotransferase; TC, cholesterol; TG, triglycerides; HDL-C, high-density lipoprotein cholesterol; LDL-C, low-density lipoprotein cholesterol.

^a^Statistical difference between gender subgroups within the same age group, *P* < 0.05;

^b^ statistical difference between different age groups of the same gender, *P* < 0.05;

^c^ statistical difference between different age groups, *P* < 0.05.

#### Distribution of serum VK levels in apparently healthy people

The levels of serum VK1, MK4, and MK7 were compared between the male and female groups in the youth group, middle-aged group, and elderly group, respectively. The Kruskal-Wallis H rank sum test results showed that there was no statistically significant difference in the three indicators between male and female subgroups in each age group (Table 2).

**Table 2.** Distribution of VK levels in males and females in different age groups of apparently healthy adults

Item	Group*	M/F (n)	Serum level P50 (P25, P75) (ng/mL)		
			Males	Females	*P value*
VK1	Young	121/154	0.604 (0.359, 0.874)	0.642 (0.423, 0.971)	0.182
	Middle-aged	22/24	0.762 (0.615, 1.066)	0.841 (0.644, 1.015)	0.895
	Elderly	72/21	0.503 (0.337, 0.728)	0.720 (0.365, 0.790)	0.254
MK4	Young	111/136	0.056 (0.04, 0.08)	0.051 (0.034, 0.08)	0.351
	Middle-aged	20/26	0.051 (0.030, 0.658)	0.048 (0.027, 0.076)	0.851
	Elderly	72/22	0.042 (0.034, 0.061)	0.02 (0.010, 0.020)	0.761
MK7	Young	132/153	0.413 (0.319, 0.558)	0.398(0.299, 0.535)	0.378
	Middle-aged	21/27	0.477 (0.400, 0.763)	0.507(0.377, 0.660)	0.640
	Elderly	62/15	0.543 (0.387, 0.687)	0.700 (0.470, 0.860)	0.054

*Definitions of age groups: Young (aged 18–44), Mid-aged (aged 45–59), Elderly (aged 60–80). Abbreviations: VK1, Vitamin K1; MK4, Menaquinone-4; MK7, Menaquinone-7; ng/ml, nanograms per millilitre.

The levels of serum VK1, MK4, and MK7 were compared among the youth group, middle-aged group, and elderly group, irrespective of gender. The Kruskal–Wallis H rank sum test results showed that the differences in the three indicators between the age groups were statistically significant (Table 3). The serum VK1 concentration in the youth group were significantly lower than those in the middle-aged and elderly groups (*P <* 0.001). The concentration of serum MK4 in the elderly group was significantly lower than that in the young and elderly groups (*P* < 0.001). The level of serum MK7 showed a significant upward trend with increase of age (*P* < 0.001).

**Table 3.** Distribution of VK levels in different age groups of apparently healthy adults

Group*	Serum level(ng/mL) P50 (P25, P75)	*P* Value
VK1 Young group (275)	0.063 (0.401, 0.912)	<0.001
Middle-aged group (46)	0.793 (0.646, 1.023)	
Elderly group (93)	0.540 (0.353, 0.781)	
MK4 Young group (247 cases)	0.053 (0.038, 0.080)	<0.001
Middle-aged group (46)	0.050 (0.030, 0.072)	
Elderly group (94)	0.038 (0.025, 0.057)	
MK7 Young group (285)	0.405 (0.308, 0.541)	<0.001
Middle-aged group (48)	0.500 (0.383, 0.662)	
Elderly group (77)	0.562 (0.402, 0.720)	

*Definitions of age groups: Young (aged 18–44), Mid-aged (aged 45–59), Elderly (aged 60–80).Abbreviations: VK1, Vitamin K1; MK4, Menaquinone-4; MK7, Menaquinone-7; ng/mL, nanograms per millilitre.

#### Establishment of serum VK reference intervals

Based on the preceding analytical outcomes, the serum concentrations of VK1, MK4, and MK7 exhibited no statistically significant variations between diverse gender groups. However, statistically significant differences were observed among various age groups. Consequently, employing non-parametric statistical methodologies, the reference intervals for VK1, MK4, and MK7 were independently calculated for each age group (Table 4).

**Table 4.** Reference intervals for distribution of VK serum levels in different age groups

Group*	Reference interval (ng/mL)		
	VK1	MK4	MK7
Young group	0.180 -1.494	0.009 - 0.115	0.169 - 0.881
Middle-aged group	0.247 - 1.446	0.002 - 0.103	0.238 - 0.936
Elderly group	0.167 - 1.445	0.003- 0.106	0.213 - 1.012

*Definitions of age groups: Young (aged 18–44), Mid-aged (aged 45–59), Elderly (aged 60–80). Abbreviations: VK1, Vitamin K1; MK4, Menaquinone-4; MK7, Menaquinone-7; ng/mL, nanograms per millilitre.

#### Verification of reference intervals

From the newly enrolled health check-up participants, twenty individuals were randomly selected from each of the age brackets: 18–44, 45–59, and over 60 years, totalling 60 participants. This sample was used to validate the newly established reference intervals for VK1, MK4, and MK7 based on age groupings. The results showed that the measurement outcomes for VK1, MK4, and MK7 in all healthy subjects were within the corresponding newly established reference intervals, fulfilling the validation criterion, which stipulated that no more than 10% of the validation samples should fall outside the reference interval. Thus, the reference intervals for VK1, MK4, and MK7 based on age groupings established in this study have undergone preliminary validation.

## Discussion

This study stratified a cohort of healthy adults from the health examinations at Guang’anmen Hospital by gender and age to compare inter-group variations. Statistical analysis revealed that serum VK levels within our study population were skewed, necessitating the use of non-parametric statistical methods to compare median levels among groups. The findings demonstrated statistically significant differences in serum VK1, MK4, and MK7 levels across the young, middle-aged, and elderly age groups. In contrast, within each age group, no significant disparities were observed between male and female subgroups. Consequently, this study utilised age-based categorization to establish reference intervals for VK1, MK4, and MK7.

In comparison with the limited domestic and international studies, the reference intervals for VK1 in all groups of this study were higher than the reported average concentration of 0.49 ± 0.40 ng/mL in European postmenopausal women.^(13)^ The lower limit of VK1 in the young group of this study, 0.180 ng/mL, is close to the previously reported lower bound of 0.17–3.05 ng/mL^(14)^ which based on healthy Italian adults, while the upper limit of 1.494 ng/mL is lower than the reported upper bound, yet both the upper and lower limits of VK1 in this study are higher than those in the 20–49-year-old healthy American adults (0.11–1.15 ng/mL).^(6)^ The lower limit of VK1 in the middle-aged group, 0.247 ng/mL, is close to the lower bound of the normal interval, with a reference interval of 0.29–2.65 nmol/L as previously described,^(15)^ and the upper limit of 1.446 ng/mL is lower than the reported upper bound.

For MK4, nearly 75% of the detected results in this study were below the normal interval (0.07–2.68 ng/mL) reported for healthy Italian adults^(14)^, and over 10% of the results were below the lower bound of the VK reference interval for women aged 18–49 reported in domestic studies.^(6)^ Similar phenomena were observed in foreign comparable studies^(16,17)^, indicating that serum MK4 concentrations are quite low and also influenced by the conversion of VK1. This suggests that MK4 requires a high sensitivity in detection methods, and the clinical application value of the established reference interval for MK4 is relatively low.

The reference intervals for MK7 established in this study, both the lower and upper limits, were significantly lower than the average concentrations reported in Japanese women of different age groups (4.96, 8.42, and 4.21 ng/mL)^(15)^, which is closely related to the widespread consumption of vitamin K-rich natto in Japan. The lower limit of the MK7 reference interval established in this study is lower than the normal adult reference interval reported in Italy (0.33–4.48 ng/mL)^(13)^, with nearly 94% of the detected values below the mean of 0.85 ng/mL reported for women of reproductive age in domestic studies,^(6)^ yet the lower limit of our reference interval is close to the lower bound of the established reference interval of 0.12–3.54 ng/mL.

Therefore, the lower bounds of the reference intervals established in this study, except for MK4, are close to those in domestic and international related studies. The differences in the high values of the upper limits are closely related to individual differences in the study population, as well as regional and dietary nutritional status. This also suggests that different regions and individuals have a relatively consistent tolerance to vitamin K deficiency, but have a higher tolerance for different serum concentrations and high doses of VK.

We acknowledge the limitations inherent in our study, which could potentially impact the reliability and applicability of the reference intervals we established. The study population was primarily drawn from ostensibly healthy individuals undergoing health checks at Guang’anmen Hospital, affiliated with the China Academy of Chinese Medical Sciences. This cohort was predominantly urban, consisting of employees and retirees from various districts of Beijing, which may introduce sampling bias when extrapolating our results to regional or national averages. Additionally, a significant number of middle-aged candidates were excluded from the study due to not meeting the inclusion criteria, leading to a limited sample size for this demographic. This underrepresentation of middle-aged participants may further bias our conclusions regarding this age group.

To address these limitations, we plan to conduct a more extensive, multicentre study that includes a broader and more diverse population, ensuring a more representative sample that can better generalise our research outcomes to the wider population.

## Conclusions

In conclusion, significant discrepancies remain in the reference intervals of VK both domestically and internationally, necessitating urgent research on VK reference levels in our country. The established reference intervals for VK1, MK4, and MK7 in this study are scientifically valid, particularly the lower limits for VK1 and MK7, which align closely with both domestic and foreign research, offering essential insights for clinical diagnosis and treatment. To address the limitations of this study, future research should consider expanding the participant pool by including individuals from rural areas and diverse age groups, along with more flexible inclusion criteria for middle-aged candidates. Implementing these strategies would enhance the demographic representation and ultimately improve the validity and clinical relevance of findings related to VK reference intervals.
